# Advancements and Innovations in Keratoconus Management: A Review of Current Practices

**DOI:** 10.3390/jcm14217491

**Published:** 2025-10-23

**Authors:** Hyeck-Soo Son, Maximilian Friedrich, Albert S. Jun, Uri S. Soiberman

**Affiliations:** 1 Department of Ophthalmology, University of Heidelberg, 69120 Heidelberg, Baden-Wuerttemberg, Germany; 2 Wilmer Eye Institute, Johns Hopkins Medical Institutions, Baltimore, MD 21287, USA; 3 Department of Ophthalmology, University of Virginia School of Medicine, Charlottesville, VA 22908, USA

**Keywords:** keratoconus, corneal ectasia, crosslinking, keratoplasty, CAIRS, bowman layer transplantation, contact lenses, ICRS, DALK

## Abstract

Keratoconus is a potentially blinding condition characterized by progressive thinning and steepening of the cornea, leading to visual impairment due to irregular astigmatism and myopia. While the exact pathophysiology is still unknown, it is believed to involve genetic, environmental, and cellular factors. Treatment options for keratoconus have significantly expanded over the past few decades. Historically, glasses were the primary means of managing mild cases, whereas rigid gas permeable contact lenses were used in moderate to advanced diseases; yet the latter were rarely tolerated by patients with steep cones, in which full-thickness corneal transplantation was often performed. However, a variety of innovative treatments have been introduced in the past decades. Corneal collagen cross-linking (CXL) has revolutionized the field by halting the progression of keratoconus through creating new covalent bonds between individual corneal fibers. Custom, soft, and scleral contact lenses have improved visual outcomes for many patients. Additionally, surgical interventions such as intrastromal ring segments and Bowman layer transplantation have provided alternatives for visual restoration and postponing a potential keratoplasty. Furthermore, innovative cellular and pharmacological KCN treatments are on the horizon, awaiting clinical trials. This review article aims to provide a comprehensive overview of the current treatment options for keratoconus.

## 1. Introduction

Keratoconus (KCN) is a common bilateral ectasia characterized by progressive corneal thinning and steepening, which may cause severe visual impairment if left untreated [1]. It primarily affects young adults, with key risk factors including eye rubbing and genetics [2].

Significant progress in diagnostics has allowed for the identification of early and subtle KCN that previously went undetected, enabling timely surgical intervention. In parallel, numerous novel therapeutic strategies have emerged—many of which were unavailable two decades ago—transforming the landscape of KCN management.

This review introduces these new treatment options that are reshaping how KCN is treated today.

## 2. Contact Lenses

Historically, spectacles or soft toric contact lenses (CL) have been the first-line management for very mild disease. However, their use is limited to early KCN, with patients requiring specialized CLs as the disease progresses.

### 2.1. Specialized Contact Lenses

Rigid gas permeable contact lenses (RGP-CLs) are commonly used as a second-line treatment for eyes with high optical aberrations. They create a smooth tear film over irregular corneas, improving visual quality [3]. However, they can cause discomfort, scarring, warpage, and may not fit well on steep keratoconic cones [4]. Larger intra-limbal RGP lenses offer better centration and comfort but require specialized fitting expertise [5].

Piggyback CLs combine a rigid lens over a soft lens to improve comfort and reduce mechanical trauma [6]. They enhance oxygen transmission with modern materials but are costly and technically complex [7]. Hybrid CLs feature a rigid center with a soft peripheral skirt, balancing clarity and comfort [8]. Studies show better patient satisfaction and vision-related quality of life compared to standard RGPs [9].

Scleral lenses are large-diameter RGP CLs that vault over the cornea, providing stable vision and comfort without touching the cornea [10]. Their design makes them ideal for advanced KCN.

### 2.2. Modern Scleral Lenses

Prosthetic Replacement of the Ocular Surface Ecosystem (PROSE) is designed using mathematical spline functions, creating seamless transitions between the optic and scleral zones [11]. The vault over the cornea is individualized for each eye, allowing improved comfort. It features fenestrations to reduce mid-day fog and special haptics to cover conjunctival masses such as pinguecula. Stason et al. analyzed 80 patients with irregular topography fitted with PROSE after having failed conventional methods and observed significant enhancement in their visual function and subjective satisfaction [12].

EyePrintPro (Advanced Vision Technologies, Lakewood, CO, USA) is another technology designed to fit precisely over the ocular surface irregularities. After taking an impression of the ocular surface, a mold is scanned with a 3D scanner and crafted with lathe technology, allowing a customized fit. While only a few studies examined its efficacy, the initial results are promising [13,14], yet it remains to be seen whether this system will be suitable for eyes with very steep cones.

## 3. Corneal Crosslinking (CXL)

CXL has revolutionized KCN management in the 21st century. First described in 1998, Seiler et al. demonstrated that applying a photosensitizing medium, riboflavin, to denuded epithelium and exposing the stroma to ultraviolet A (UV-A) light can stiffen the anterior cornea while maintaining its clarity [15]. The same group later showed that the treatment can prevent KCN progression [16].

The exact mechanism of CXL remains ambiguous, but it is generally accepted that the treatment induces new covalent bonds between adjacent collagen fibers rather than strengthening individual fibrils [17]. Under UV-A irradiation, riboflavin acts as a photosensitizer, generating reactive oxygen species such as singlet oxygen and superoxide radicals. These radicals facilitate the formation of covalent bonds between collagen amino acids and proteoglycan core proteins, enhancing inter-fibrillar connectivity and increasing corneal stiffness and enzymatic resistance.

### 3.1. Conventional Protocol

The “Dresden protocol” involves topical administration of riboflavin solution (0.1% riboflavin in 20% dextran) to denuded stroma every 2 min for 30 min, after which UV-A (365–370 nm) is irradiated at 3.0 mW/cm^2^ for 30 min to reach a total surface dose of 5.4 J/cm^2^ [15]. During irradiation, riboflavin is applied to the cornea every 5 min. Numerous studies have since shown the safety and efficacy of this protocol in stabilizing the cone and limiting visual impairment [18,19].

### 3.2. Accelerated Protocols

The Bunsen–Roscoe law of reciprocity states that the same photochemical effect can be achieved with less illumination time if the irradiation intensity is increased [20]. Consequently, “accelerated” CXL (A-CXL) protocols were proposed to shorten the procedure [20]. Examples include irradiating 9–12 mW/cm^2^ for 9–10 min for a total irradiance of 5.4 J/cm^2^ or 30 mW/cm^2^ for 3 min for a total irradiance of 7.2 J/cm^2^, either epi-on or epi-off [21,22,23,24,25]. Conflicting reports have been published regarding their efficacy. One meta-analysis demonstrated that both A-CXL and standard protocols attain comparable safety and efficacy in hindering KCN progression and improving vision at 1-year postoperatively [26]. Contrarily, another meta-analysis observed a greater flattening effect and deeper demarcation lines after the Dresden protocol vs. the accelerated protocol in progressive KCN eyes [27]. Furthermore, a recent study observed higher rates of disease progression after accelerated CXL of 9 mW/cm^2^ for 10 min in eyes with Kmax of >58D or in patients <19 years of age, suggesting potentially favorable outcomes with the conventional protocol in these cohorts [28]. Randomized controlled studies comparing all published accelerated protocols may also provide more insight into their efficacy and safety.

For effective treatment, sufficient oxygen availability is necessary, and it is hypothesized that high radiation intensity used in A-CXL protocols may cause rapid oxygen depletion that exceeds the rate of oxygen replenishment, reducing the stiffening efficacy [29].

### 3.3. Pulsed Crosslinking

Consequently, accelerated but “pulsed” CXL was proposed to improve oxygen delivery by allowing episodes of oxygen replenishment in corneal stroma. Mazzotta et al. compared the efficacy of continuous- vs. pulsed-accelerated CXL protocols in 20 KCN patients with progression and found that the latter led to improved functional outcomes with comparable safety, likely due to higher oxygen levels maintained during intermittent UV-exposure [30]. Numerous studies have also shown the efficacy of the pulsed protocol in stabilizing ectasia progression [24,31,32]. A recent meta-analysis found pulsed CXL to be non-inferior to the continuous protocol in improving visual and keratometric outcomes in KCN [33]; however, heterogenous study designs and non-randomized studies were included, which may undermine the study’s validity. Future randomized studies with larger sample sizes are necessary to establish the superiority of pulsed CXL.

### 3.4. Epithelium-On Protocols

Epi-off protocols carry risks of infection, pain, haze, and persistent epithelial defects [34]. Consequently, epithelium-on protocols have been described where ethylenediaminetetraacetic acid (EDTA) or benzalkonium chloride (BAC) is administered to the epithelium to enhance its permeability, allowing sufficient stromal diffusion of riboflavin. However, the transepithelial technique was found to be potentially less efficacious [35,36] because the epithelium acts as an anatomical barrier for the penetration of oxygen and solutes (such as riboflavin) and may consume more oxygen than the stroma, limiting sufficient oxygen availability [37]. Providing supplemental oxygen during the epi-on procedure may enhance its outcomes [38].

### 3.5. Iontophoresis-Assisted Crosslinking

Iontophoresis-assisted CXL is based on the Nernst–Planck effect, whereby charged particles follow the electrical current and penetrate tissues. Given the potential risks of epi-off protocols, iontophoresis-assisted CXL was introduced to allow transepithelial electromigration [39]: after placing a ring electrode on the cornea, filled with ionized riboflavin, a current generator is activated to deliver riboflavin into the stroma [40]. In a meta-analysis, Wan et al. found the iontophoresis-assisted CXL to be equally efficacious in improving visual, topographic, and aberrometric outcomes compared to the Dresden protocol [41]. Epi-off iontophoresis-assisted CXL has also been shown to provide corneal stabilization in mild to moderate KCN, with shorter procedural time than the epi-on protocol [42]. However, there is limited long-term data, with some studies observing inferiority to the conventional protocol and disease progression in 26% of treated eyes at 7 years postoperatively [43].

### 3.6. Crosslinking in Thin Corneas

CXL has limited applicability in thin corneas with stromal thickness of <400 µm after saturation with the chromophore/photoactivator, as UVA irradiance may cause endothelial damage [44]. However, often the very patients who need the CXL are the same ones with advanced KCN declared ineligible for CXL due to too-thin corneas. Thus, various modified protocols have been proposed to allow safe CXL in such eyes.

Customized pachymetry-guided epithelial removal involves selective removal of the epithelium only over the thinnest area while preserving it peripherally, allowing better UVA absorption where needed and reducing exposure to the endothelium [45]. Early data suggest improved safety and targeted stiffening, but long-term efficacy data are limited [46].

Alternatively, hypoosmolar riboflavin (0.1%) can be instilled for 10–30 min to induce stromal swelling, increasing stromal thickness temporarily above 400 μm before irradiation. Hafezi et al. observed pachymetric increase of up to 100 µm, achieving effective CXL while protecting the endothelium [47].

Jacob et al. described using UV barrier-free soft CLs pre-soaked in iso-osmolar riboflavin to artificially thicken the stroma by approximately 100 µm [48]. While the 2-year results in progressive KCN eyes seem promising, with no adverse events [49], few studies observed less stiffening effect amongst eyes treated with CL-assisted CXL vs. the standard protocol [50,51].

Sachdev et al. placed stromal lenticules removed from patients undergoing small-incision lenticule extraction (SMILE) for myopic correction over the cone to increase the stromal thickness [52]. The authors noted corneal stability by 6 months postoperatively with no significant endothelial damage. However, additional efforts associated with serological testing and storage of lenticule tissues may hinder widespread adoption [53].

Lastly, applying UVA at a fixed intensity of 3 mW/cm^2^ while adjusting the irradiation time to intraoperative stromal thickness can achieve effective CXL while avoiding endothelial damage [54]. Termed the “Sub400 Protocol”, the authors successfully treated corneas as thin as 214 µm of stroma and observed disease stabilization in 90% of progressive KCN corneas at 1 year with no signs of endothelial decompensation.

### 3.7. Customized Crosslinking

In topography-guided customized CXL, the irradiation zone is not uniform across the anterior surface but modified to multiple concentric circular zones centered on the cone to allow a graded treatment pattern. In nine eyes with progressive KCN, Sachdev observed greater topographic flattening effects and regularization vs. conventional CXL, resulting in CDVA improvement [55]. Despite promising initial results [56], long-term outcomes in a larger sample size are necessary to determine their benefit.

Further customized protocols are currently underway to offer tailored treatment.

### 3.8. Crosslinking Plus

Crosslinking-Plus (CXL-Plus) describes an adjunctive use of refractive procedures such as photorefractive (PRK) or phototherapeutic keratectomy (PTK) to not only stop progression but also improve vision. While there is no global consensus on the ideal candidate for CXL-Plus, this approach is generally recognized as more suitable for eyes with mild to moderate KCN with potential for visual improvement and sufficient stromal thickness for ablation (if combined with PRK).

Kymionis et al. demonstrated that performing simultaneous topography-guided PRK can attain significant improvements in CDVA, spherical equivalent, and keratometry in progressive KCN eyes [57]. Another study found this approach to be superior to the sequential approach, as the laser ablation does not interfere with crosslinked stromal fibers, minimizing the risk of stromal scarring while increasing treatment efficacy [58]. A recent meta-analysis has also found simultaneous CXL with customized PRK to deliver superior visual outcomes on progressive KCN eyes compared to performing CXL alone [59].

Some studies have also demonstrated that performing PTK for epithelial debridement may better regularize the irregular topography by offering a highly uniform and predictable depth of removal, achieving marked visual and topographic improvements [60,61].

The past decades have seen an explosion of novel CXL protocols (Supplementary Table S1). Although CXL can stabilize KCN in all age groups and improve visual and morphologic parameters [62,63], there is a dearth of randomized controlled trials comparing different protocols. Furthermore, most studies do not clearly define whether the eyes had progressive or stable disease, complicating a direct comparison. Lastly, 22% of pediatric KCN eyes have been found to show disease progression despite undergoing CXL [64], highlighting the need for long-term follow-up studies in case a re-treatment becomes necessary.

## 4. Intrastromal Implants

### 4.1. Intracorneal Ring Segments (ICRS)

Composed of polymethylmetacrylate (PMMA) material, ICRS are rigid, crescent-shaped implants that are inserted into the stroma to regularize the topography. Once implanted into a manual or femtosecond laser-created tunnel, ICRS redistribute the corneal stress forces, flattening its steep areas and reducing irregular astigmatism [65]. The procedure is minimally invasive and reversible, allowing easy removal if necessary. Combining CXL with ICRS has also been suggested to aid in arresting KCN [66]. However, ICRS has been associated with extrusion, corneal melting, infectious keratitis, neovascularization, and Descemet membrane (DM) perforation, which may require explantation [67]. Furthermore, as ICRS need to be implanted close to the pupillary axis to exert their effects, aesthetic complaints and halo perception have been reported [68].

### 4.2. Corneal Allogeneic Intrastromal Ring Segments (CAIRS)

In 2017, Jacob et al. proposed using human corneal allogeneic tissues instead of PMMA to mitigate the PMMA-associated complications of ICRS [69]. Similar to ICRS transplantation, CAIRS transplantation involves manual or femtosecond laser-assisted creation of stromal pockets into which the segments are inserted to induce central flattening and topographical regularization. Various studies have shown that CAIRS achieve visual, refractive, and tomographic improvements, with no PMMA-related complications observed with ICRS, highlighting their favorable complication profile [70,71]. Another advantage of CAIRS lies in their unlimited potential of customizability: depending on the cone location and keratometry gradation, the size and length of CAIRS can be fully tapered, leading to optimal outcomes for each patient [72].

Another similar procedure is Corneal Tissue Addition for Keratoplasty (CTAK). Similar to CAIRS, in CTAK, allogeneic corneal stromal rings are implanted into a stromal pocket to regularize the central cornea and flatten the cone [73]. However, in CTAK, tissue prepared according to a proprietary nomogram (CorneaGen, Seattle, WA, USA) is used.

While different surgeon-developed nomograms for CAIRS have been proposed, most studies reported to date are case series with relatively short follow-up; thus, large-sample analyses with longer follow-ups are necessary to investigate the accuracy and reproducibility of the nomograms for various KCN phenotypes [74].

## 5. Corneal Transplantation

### 5.1. Penetrating Keratoplasty (PKP)

Historically, PKP was the standard care for patients with moderate to severe cones who could not tolerate RGP lenses; it replaced the diseased cornea and improved topography, providing visual rehabilitation with a high graft survival rate [75]. However, given its open-sky procedural nature, it bears potential risks of suprachoroidal hemorrhage and extrusion of intraocular contents.

### 5.2. Deep Anterior Lamellar Keratoplasty (DALK)

DALK is a partial-thickness keratoplasty where DM and endothelium are not replaced, mitigating the risk of endothelial rejection, as opposed to PKP [76]. While DALK and PKP both achieve similar long-term visual outcomes for KCN, the former yielded less endothelial cell loss and incidence of ocular hypertension, providing compelling evidence for its favorable safety profile [77].

One of the most well-known DALK techniques is the big-bubble (BB) approach [78]. However, its success rates are challenged by the technical difficulty involved in creating a proper BB [78]. Furthermore, a recent analysis demonstrated that advanced KCN with steeper and more ectatic cones are more prone to BB failure [79].

Consequently, various recipient preparation methods have been proposed, such as the femtosecond laser-assisted DALK, which may reduce the rate of intraoperative DM perforation and conversion to PK compared to manual DALK [80]. Yet, it requires the use of the laser, increasing the procedure cost. Alternatively, the peel-off techniques [81,82] have been shown to be reproducible and effective in reaching the deep stromal plane.

## 6. Stromal Keratophakia

### 6.1. Bowman Layer Transplantation (BLT)

In 2014, Melles et al. described inserting an isolated Bowman layer (BL) into the mid-stromal pocket in advanced KCN eyes [83]. Given the putative role of BL in corneal biomechanical stability, BLT is postulated to aid in stabilizing the cone and postponing keratoplasty [84]. Indeed, notable visual and refractive improvements were observed after inlay-BLT with a significant decrease in maximum keratometry [85]. However, manual preparation of a stromal pocket within a thin cornea is technically challenging, and while a femtosecond laser can be used for a more precise preparation [86], it is not inexpensive and ubiquitous in all practices. Inlay BLT has also been associated with rare instances of sterile corneal necrosis and postoperative hydrops [87]. Later, the same group presented an “onlay”-BLT technique, where the BL is grafted onto the corneal stroma after epithelial debridement, achieving up to −5 D of corneal flattening in progressive KCN eyes [88]. Kocaba et al. observed that KCN eyes with preoperative Kmax of >69D experienced significant decreases in Kmax, while those with preoperative Kmax of <69D did not, demonstrating that advanced cones may be more likely to benefit from the onlay-BLT [89]. While promising, a widespread adoption of the BLT may be hindered by the technical difficulty of obtaining an isolated BL graft [90].

### 6.2. Stromal Lenticule Addition Keratoplasty (SLAK)

SLAK involves implanting a customized lenticule into a femtosecond-laser-created stromal pocket to restore corneal thickness and improve topography. These lenticules may be derived from cadaveric corneas or extracted from myopic patients undergoing SMILE surgery [91]. The shape and thickness of the lenticule can be tailored to the patient’s specific corneal topography and disease severity, with forms ranging from concave and convex to donut, ring, or asymmetric shapes.

SLAK is often performed in moderate to advanced KCN eyes with progressive disease or CL intolerance, and studies have thus far demonstrated consistent improvements in corneal thickness, keratometry, and vision [92,93]. Importantly, no cases of lenticule rejection or permanent scarring were reported in follow-up periods up to five years, indicating safety and biocompatibility [94].

However, concerns remain regarding surgical complexity, lenticule availability, and variability in biomechanical outcomes. Lenticule banks and cryopreservation methods are being explored to address the shortage of suitable donor tissues [95]. Combining SLAK with corneal CXL may enhance biomechanical stability, though further evidence is needed.

### 6.3. Bandage Therapeutic Optical Keratoplasty (BTOK)

BTOK is an interlamellar corneal grafting technique developed in 2013 for the surgical management of progressive stage II–III keratoconus. The procedure involves implantation of a customized donor allograft containing Bowman’s and stromal layers into a femtosecond-laser–created intrastromal pocket to thicken and biomechanically reinforce the ectatic cornea. In a prospective study of 126 eyes, BTOK led to significant and stable improvements in uncorrected and corrected visual acuity, a reduction in maximum keratometry values by up to 8 D, and slight increases in minimal corneal thickness over five years, with no reported cases of graft opacity or rejection [96]. Further research is required to compare their efficacy compared to other stromal keratophakic procedures.

The selection criteria, outcomes, and risks of the abovementioned keratoplasties are summarized in Table 1.

## 7. Future Perspectives

Today, various novel trials are underway for innovative treatment approaches. One study assessed the potential of autologous adipose-derived adult stem cells (ADASCs) in corneal stromal regeneration [102]. ADASCs can differentiate into keratocytes, are easily accessible via liposuction, and demonstrate high cell retrieval efficiency, proving to be a promising source of autologous stem cells. Upon injection of ADASCs into the stromal pocket of advanced KCN eyes, signs of new collagen production were observed at the level of the stromal pocket using a confocal biomicroscope, along with a mean increase of 16.5 µm in central corneal thickness and vision improvement with no complications, demonstrating the potential of ADASCs as a cellular therapy for KCN.

Other studies investigated the roles of WNT10A and collagen XII in the pathogenesis of KCN. Wnt signaling regulates extracellular matrix production and collagen XII production in corneal epithelium; collagen XII is postulated to support stress-bearing areas, so its depletion is likely to play a significant role in the biomechanical instability seen in KCN [103]. Furthermore, mechanical strain, such as eye rubbing, causes a marked reduction in protein expression of these molecules, providing a molecular link between chronic eye rubbing, WNT10A dysregulation, and biomechanical failure in KCN [104]. Thus, the Wnt pathway presents a potential therapeutic target for molecular KCN treatment.

Lastly, IVMED-80 (iVeena, Salt Lake City, UT, USA) was recently granted orphan drug designation by the U.S. Food and Drug Administration. Its formulation is based on a cofactor for lysyl oxidase (LOX) activity, which is involved in the formation of collagen crosslinks and, thus, natural corneal stiffening. With LOX dysregulation being implicated in KCN pathogenesis, with resultant reduction in biomechanical stability [105], IVMED-80 aims to increase LOX activity in KCN eyes to induce corneal stiffening. In a randomized, placebo-controlled, masked pilot clinical study consisting of 36 moderate KCN patients, about 1D of Kmax reduction was observed at 16 weeks upon IVMED-80 instillation vs. 0.46D of Kmax increase in the placebo group at 16 weeks, showing encouraging results of this novel topical KCN therapy [106].

## 8. Conclusions

The past two decades have brought a rapid expansion in KCN treatment options. Innovations in CL technology, surgical techniques, and CXL protocols have enabled more personalized, stage-specific care. Nevertheless, there is a pressing need for additional studies to better define patient selection criteria, establish long-term outcomes, and develop standardized nomograms. Until such evidence is available, treatment decisions should be made on a case-by-case basis with careful consideration of individual patient characteristics and expectations.

## Figures and Tables

**Table 1:** Comparison of selection criteria and outcomes of keratoplasties for keratoconus treatment.

Surgical Intervention	Selection Criteria	BCVA Outcome	Complications/Risks
Intracorneal Ring Segments (ICRS)	Clear Cornea and Pachymetry at implantation site >400 µm and mild-to-moderate KCN +/− CL intolerance	Improvement by 1–2 (up to 4) lines [96]	Glare, extrusion, corneal melting, infectious keratitis, neovascularization, DM perforation
Corneal Allogeneic Intrastromal Ring Segments (CAIRS)/Corneal Tissue Addition for Keratoplasty (CTAK)	Clear Cornea & Pachymetry at implantation site >350 µm and mild-to-moderate KCN +/− CL intolerance	Mean improvement by 4 lines [74]	One report of acute rejection, segment subluxation or extrusion
Penetrating Keratoplasty (PKP)	Central corneal scars or Severe KCN	Over 90% with BCVA of 0.3 logMAR [97,98,99]	Irregular astigmatism, graft rejection, ECL, ocular hypertension, intraoperative suprachoroidal hemorrhage and extrusion of intraocular contents
Deep Anterior Lamellar Keratoplasty (DALK)	Central corneal scars or Severe KCN	Mean BCVA: 0.29 ± 0.24 logMAR after one year [77]	Irregular astigmatism, less ECL and rejection rate than PKP, difficult technique, intraoperative conversion to PKP due to DM rupture
Bowman Layer Transplantation (BLT)	Clear Cornea and Severe KCN ineligible for CXL or ICRS/CAIRS	Mean improvement by 0.29–0.37 logMAR [100,101]	Sterile corneal necrosis, postoperative hydrops, KCN progression, intraoperative DM perforation
Stromal Lenticule Addition Keratoplasty (SLAK)	Clear Cornea and Severe KCN ineligible for CXL or ICRS/CAIRS and TCT > 300 µm	Mean improvement by 0.169 logMAR [91]	Difficult technique, haze, transient corneal edema, anterior corneal surface perforation
Bandage Therapeutic Optical Keratoplasty (BTOK)	Clear Cornea and Severe KCN ineligible for CXL or ICRS/CAIRS and Progression	Mean BCVA: ~0.7 decimal after one year [96]	Difficult technique, transient corneal edema, ectasia progression

CL = Contact Lens; CXL = crosslinking; DM = Descemet Membrane; ECL = Endothelial Cell Loss; KCN = Keratoconus; TCT = Thinnest Corneal Thickness.

## Supplementary Materials

The following supporting information can be downloaded at: https://www.mdpi.com/article/10.3390/jcm14217491/s1, Table S1. Comparison of Corneal Crosslinking (CXL) Protocols.
