# Supplementary material for: Advancements and Innovations in Keratoconus Management: A Review of Current Practices
Source: J Clin Med. 2025 Oct 23;14(21):7491. doi: 10.3390/jcm14217491 (PMC12609086; doi:10.3390/jcm14217491)
Supplement: Supplementary file 1 [file jcm-14-07491-s001.zip › jcm-3910330-supplementary.pdf]

Supplementary Table S1. Comparison of Corneal Crosslinking (CXL) Protocols.

Protocol	Indications	Advantages	Disadvantages
**Standard (Dresden) CXL (3 mW/cm^2, 30 min)**	Keratoconus, corneal ectasia in corneas ≥400μm	Well-studied, effective, long-term stability	Long treatment time, not suitable for corneas <400μm, risks associated with epithelial removal
**Accelerated CXL (9-30 mW/cm^2, 3-10 min)**	Mild keratoconus, ectasia	Shorter treatment time, improved patient comfort	Reduced efficacy due to oxygen depletion, may be less effective in advanced cases due to shallower crosslinking, risks associated with epithelial removal
**Pulsed and High-Flunece CXL**	Moderate to mild keratoconus	Allows oxygen replenishment, potentially deeper crosslinking	Long-term safety still under investigation, risks associated with epithelial removal

**Transepithelial (Epi-On) CXL**	Patients intolerant to epi-off CXL, mild cases	Less pain, faster recovery	Reduced riboflavin penetration, may have lower efficacy compared to epi-off CXL
**Iontophoresis-Assisted CXL**	Mild to moderate keratoconus, patients intolerant to epi-off CXL	Faster riboflavin absorption, less pain, avoids epithelial removal, shorter recovery time	Lower riboflavin penetration, reduced efficacy compared to epi-off CXL, may not be suitable for advanced keratoconus, requires special instruments
**Epithelial Island CXL**	Stromal thickness <400μm	Protects thinnest corneal regions from excessive UV exposure	Uneven crosslinking, risk of irregular outcomes, difficulty in outlining the exact area of thinning when removing the epithelium

**Hypo-Osmolar Riboflavin CXL**	Stromal thickness <400μm needing temporary thickening	Increases stromal thickness to allow treatment	Variable swelling response, ineffective in very thin corneas
**Contact Lens-Assisted CXL**	Stromal thickness <400μm	Allows treatment of thin corneas	Reduced oxygen availability, lower efficacy, cannot be used in extremely thin corneas
**Sub400 Protocol**	Stromal thickness <400μm, severe keratoconus	Safe for ultrathin corneas, standardized	Relies heavily on accurate intraoperative pachymetry, long-term results are unknown
**Topography-Guided CXL**	Irregular corneas, post-LASIK ectasia	Customizes treatment based on individual corneal maps	More complex, needs advanced imaging and planning
**Athens Protocol (CXL + Topography-Guided PRK)**	Progressive keratoconus with	Improves vision while stabilizing the cornea,	Risk of corneal haze, requires careful patient selection and thin

	significant refractive error	reduces irregular astigmatism	corneas cannot be treated, risk of progression
**Cretan Protocol (Sequential CXL and PRK)**	Keratoconus with refractive error	PRK performed **after** CXL to allow corneal stabilization first and optimization of corneal shape	Longer treatment process, delayed visual improvement, risk of progression
**Tel Aviv Protocol (CXL + ePRK)**	Keratoconus with significant ectasia, corneal thickness ≥400μm	Combines topographic correction with CXL; improves vision and stabilizes cone	Not for thin corneas, risks include haze, delayed epithelial healing

CXL = crosslinking; PRK = photorefractive keratectomy; ePRK = epithelial photorefractive keratectomy
